# 566. Optimization of Voriconazole Target Trough Attainment via Utilization of Therapeutic Drug Monitoring

**DOI:** 10.1093/ofid/ofaf695.175

**Published:** 2026-01-11

**Authors:** Donna Wesslen, Rupal Jaffa, Ethan Rausch, Jennifer A Schweiger

**Affiliations:** Advocate Health: Atrium Health Antimicrobial Support Network, Charlotte, NC; Advocate Health: Atrium Health Antimicrobial Support Network, Charlotte, NC; Advocate Health: Atrium Health Antimicrobial Support Network, Charlotte, NC; Advocate Health: Atrium Health Antimicrobial Support Network, Charlotte, NC

## Abstract

**Background:**

Voriconazole therapeutic drug monitoring is recommended to minimize toxicity and ensure adequate drug exposure. However, data regarding the optimal approach to dose adjustments for levels outside the therapeutic range are limited.

**Table 1.**
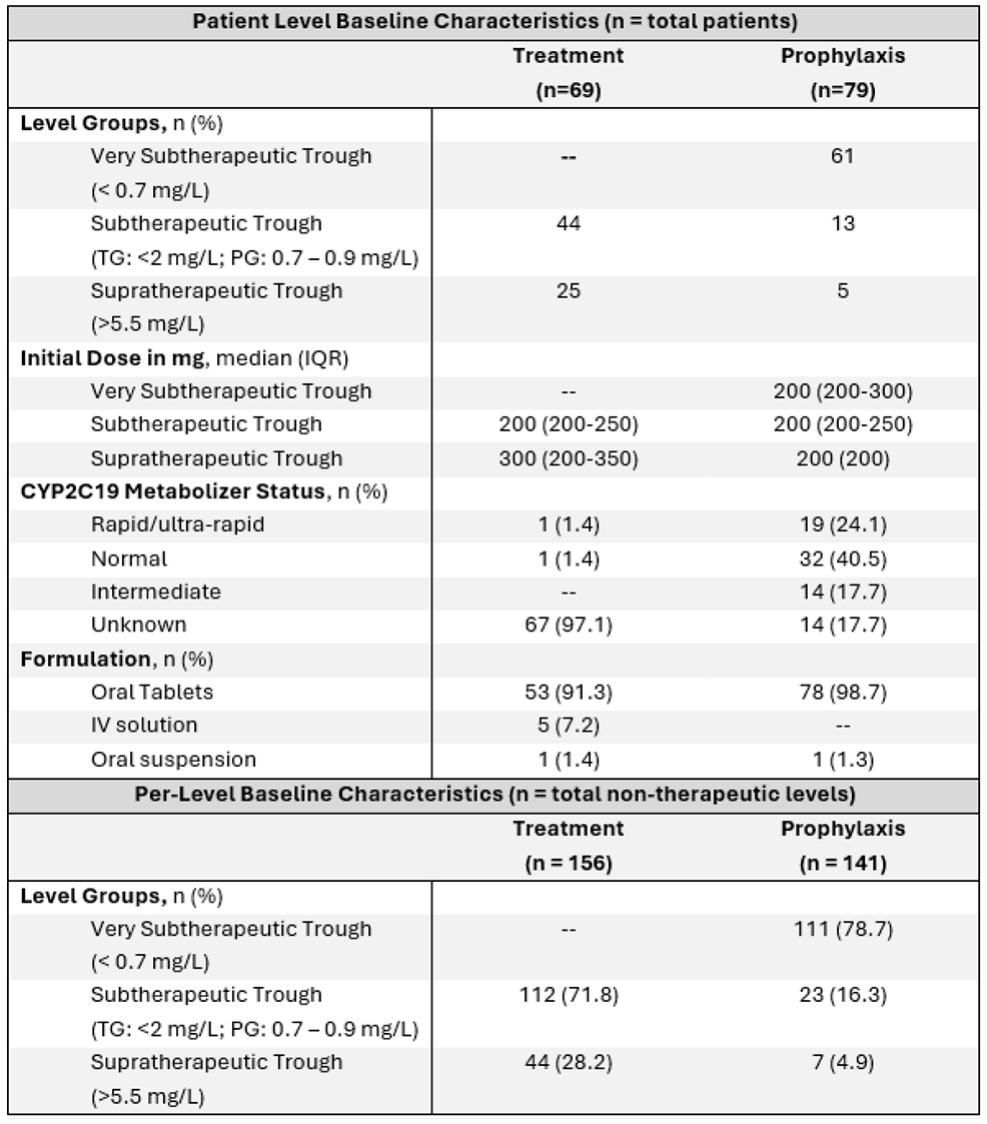
Voriconazole Characteristics

**Figure:**
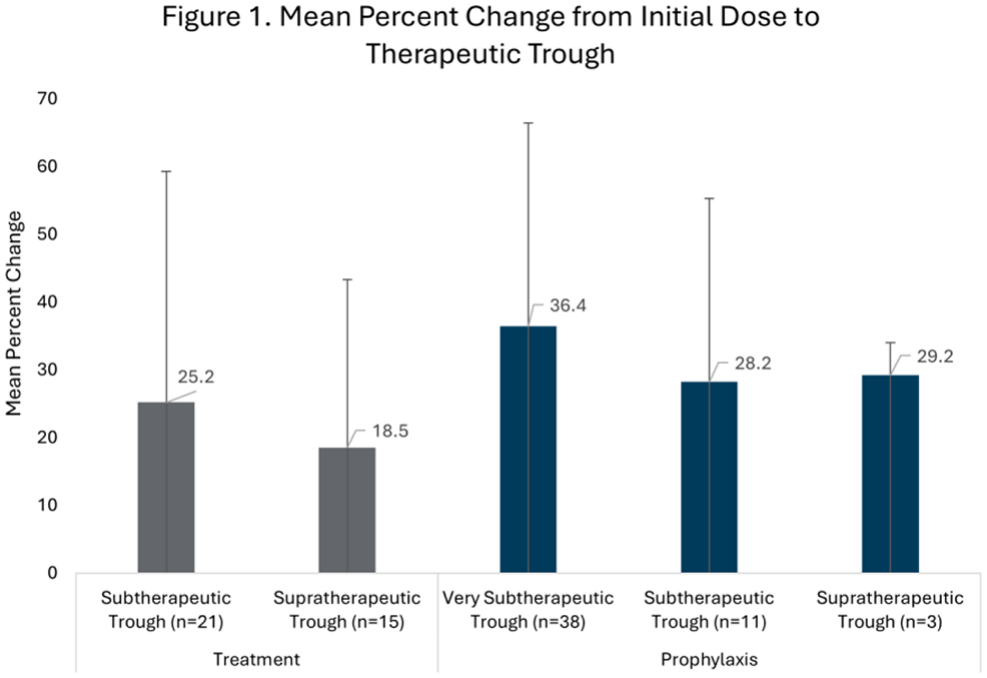

**Methods:**

This was a multicenter, retrospective, single health system study of adult patients with voriconazole levels outside the therapeutic range collected from April 2021 to August 2024. Data, including baseline characteristics and voriconazole prescribing, were collected on a per patient and per level basis. Patients were categorized by indication into groups – treatment group (TG) with therapeutic trough (TT) 2 – 5.5 mg/L or prophylaxis group (PG) with TT 1 – 5.5 mg/L. Patients and levels were further classified into level groups (Table 1). The primary outcome was mean percent dose change required to achieve (TT) in the TG or PG by level group. Secondary outcomes included dose change stratified by formulation and CYP2C19 phenotype; number of days and dose adjustments to achieve TT; a per-level analysis of provider response rates to non-therapeutic troughs by level group; and per-level rates of TT attainment based on level group and dose adjustment strategy (no adjustment, increase, decrease).

**Figure:**
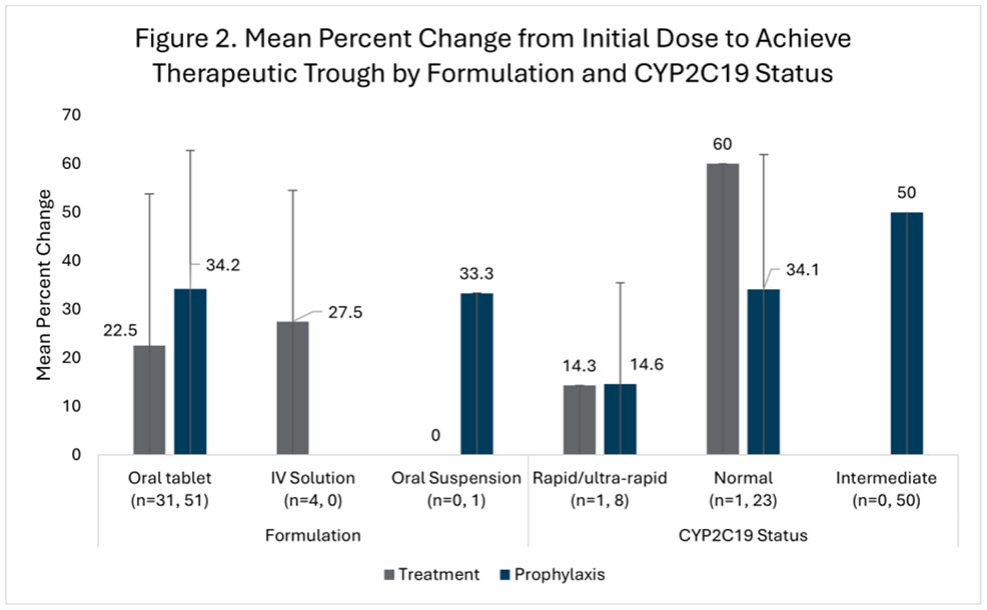

**Table 2.**
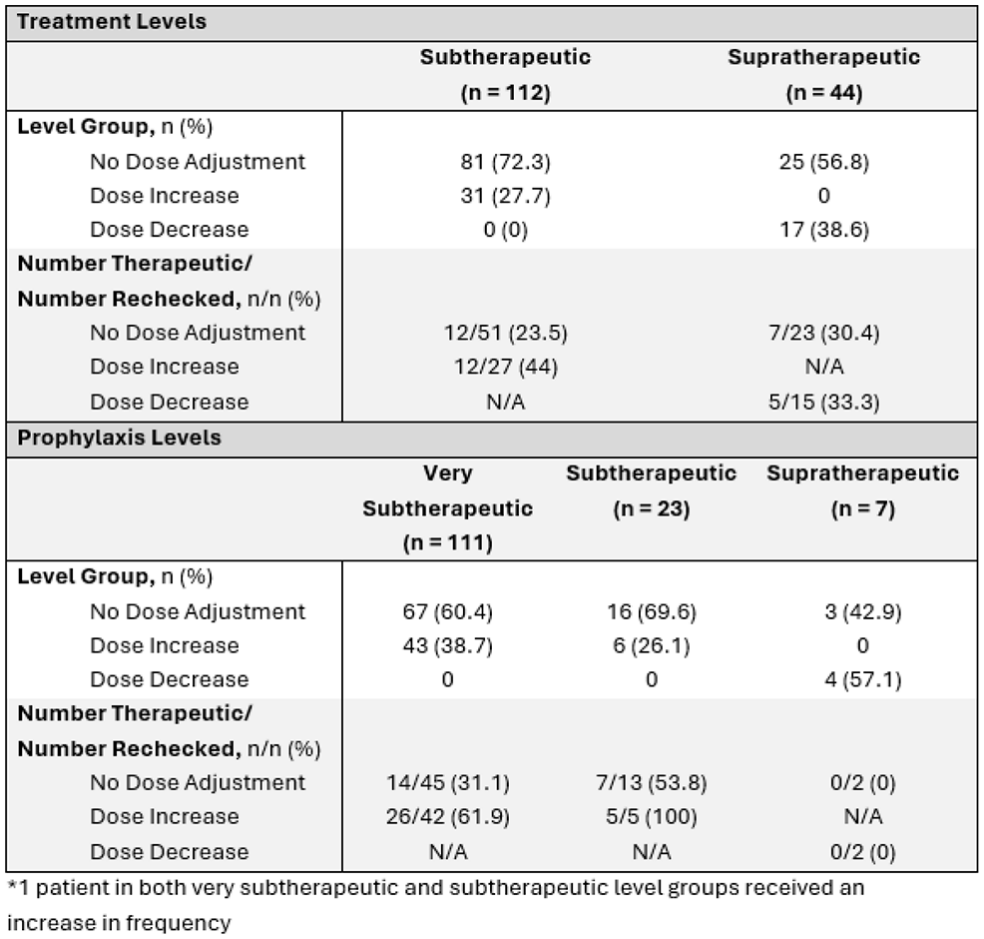
Effect of Dose Adjustment on TT Attainment

**Results:**

A total of 148 patients (69 TG, 79 PG) and 297 levels (156 treatment, 141 prophylaxis) were included (Table 1). Mean percent dose change to reach TT is available in Figure 1. No differences in required dose adjustments based on formulation were noted. Rapid/ultra-rapid CYP2C19 metabolizers required smaller dose adjustments than other phenotypes (Figure 2). Rates of dose adjustment and TT attainment are available in Table 2. For very subtherapeutic patients in PG, dose adjustment increased odds of TT attainment (OR 3.59, 95%CI 1.72-8.73).

**Conclusion:**

Initial level group and dose formulation did not impact required dose adjustments to achieve TT in the TG or PG. A small patient population precludes definitive conclusions on the impact of metabolizer status. Rates of level recheck, and dose adjustment were low, but this study suggests that adjustment results in a higher likelihood of TT than monitoring without adjustment and that an adjustment of 30% for any non-therapeutic level may be appropriate.

**Disclosures:**

All Authors: No reported disclosures

